# Review of the Characteristics of School-Based Integrated Mental Health and Substance Use Prevention Programs for Adolescents Evaluated Through Clinical Trials Between 2016 and 2026

**DOI:** 10.3390/children13091139

**Published:** 2026-08-25

**Authors:** Kyung-Young Hong, Jihye Shin

**Affiliations:** 1 College of Nursing, Eulji University, Uijeongbu 11759, Republic of Korea; hong2813@eulji.ac.kr; 2 College of Nursing, Catholic University of Pusan, Busan 46252, Republic of Korea

**Keywords:** adolescence, mental health, substance-related disorders, school-based prevention, help-seeking behavior, life skills, integrated prevention

## Abstract

**Highlights:**

**What are the main findings?**

- Fifteen studies representing ten unique school-based integrated prevention programs for adolescents were identified.
- Mental health literacy, emotional regulation, coping skills, and help-seeking promotion were the most common mental health components.
- Drug literacy, resistance skills, alcohol prevention, and cannabis prevention were the most frequently incorporated substance use prevention components.

**What are the implications of the main findings?**

- The programs included in this review combined substance use prevention with one or more mental health promotion components.
- The included programs used varied delivery approaches and incorporated psychosocial skill development, peer support, and, less frequently, family-focused components.
- The identified program characteristics may inform the design, implementation, and evaluation of integrated school-based prevention programs.

**Abstract:**

Background/Objectives: Adolescent substance use and mental health problems frequently co-occur and share common risk and protective factors. Some school-based prevention programs address substance use and mental health within an integrated approach. However, the characteristics and intervention components of these integrated approaches remain insufficiently understood. This review aimed to describe the program characteristics of school-based integrated mental health and substance use prevention programs for adolescents that were evaluated in randomized or cluster randomized controlled trials (RCTs/cRCTs), with particular attention to their intervention components, delivery approaches and prevention strategies. Methods: This review used a structured approach comprising a systematic literature search, predefined eligibility criteria, independent study selection, standardized data extraction and coding, and descriptive synthesis. Electronic databases (PubMed, Embase, Cochrane Library, CINAHL, PsycINFO, KMbase, KoreaMed, ScienceON, DBpia, KISS, RISS, and PQDT) were searched for studies published between January 2016 and April 2026. Studies were eligible if they were randomized or cluster randomized controlled trials evaluating school-based interventions that incorporated both substance use prevention and mental health promotion components among adolescents. Data were extracted and synthesized descriptively at the program level to summarize program characteristics and intervention components. Results: Fifteen studies representing 10 unique programs were included. Mental health literacy was identified in 5 programs and emotional regulation in 4. Drug literacy and resistance skills were each identified in 6 programs, alcohol prevention in 5, and cannabis prevention in 4. A descriptive cross-domain analysis identified overlapping patterns involving literacy, self-regulation, help-seeking, social competence, peer support, and family-focused approaches rather than mutually exclusive program types. Conclusions: The school-based integrated prevention programs evaluated in randomized or cluster randomized controlled trials and included in this review combined substance use prevention with mental health promotion components, including psychosocial skill development, peer support, and, in one program, a family-focused approach. The descriptive synthesis of program characteristics provides an initial basis for understanding the diverse approaches used in integrated school-based prevention and may inform the future design and evaluation of programs targeting adolescent mental health and substance use.

## 1. Introduction

Adolescence is a critical developmental period characterized by substantial biological, psychological, and social changes [1]. During this period, many mental health problems first emerge, including depression, anxiety, emotional distress, and behavioral difficulties [1]. At the same time, adolescents may begin experimenting with alcohol, tobacco, cannabis, and other substances [2,3]. Substance use during adolescence is associated with adverse physical, psychological, and social outcomes [4,5], as well as poor academic performance, interpersonal difficulties, increased healthcare utilization, and longer-term health risks [6,7]. Mental health problems and substance use frequently co-occur among adolescents and share several risk and protective factors, including emotional dysregulation, maladaptive coping, impulsivity, peer influences, poor problem-solving abilities, and barriers to help-seeking [8,9,10]. Adolescents experiencing psychological distress may be more likely to engage in substance use as a maladaptive coping mechanism [11,12], whereas substance use may exacerbate emotional and behavioral problems [13]. This reciprocal relationship provides a rationale for prevention approaches that address shared underlying mechanisms rather than treating mental health and substance use as entirely separate concerns [14]. Historically, school-based substance use prevention programs primarily focused on providing information about the risks and consequences of alcohol, tobacco, and other drug use [15,16].

Although knowledge-based approaches remain important, information alone is often insufficient to produce sustained behavioral change [17]. Some school-based programs have therefore incorporated mental health promotion components, including mental health literacy, coping skills, emotional regulation, cognitive behavioral strategies, communication and social skills, peer support, decision-making skills, and help-seeking promotion [18,19,20]. These components are intended to address risk and protective mechanisms relevant to both mental health and substance use [21,22].

Schools provide an important setting for prevention because they enable access to large numbers of adolescents during a critical developmental period. School-based interventions may use universal, selective, or indicated prevention strategies [23] and can be incorporated into existing educational and health-promotion systems. Schools also provide opportunities to strengthen psychosocial competencies, foster supportive peer relationships, engage families, and facilitate access to mental health resources [24,25]. Examples of integrated school-based programs have been reported in specific national contexts, including Australia, Switzerland, and Brazil [19,26,27,28]. However, these examples do not establish how widely or consistently such programs are available or implemented across countries.

Previous reviews have primarily examined intervention effectiveness, substance use outcomes, or specific prevention approaches [14,16,17]. Although these reviews provide important evidence regarding intervention outcomes, they have not systematically distinguished between the structural and implementation-related characteristics of integrated programs and the specific intervention components delivered within them. Moreover, limited attention has been given to how mental health promotion and substance use prevention components are combined within individual programs. The present review systematically identified school-based integrated mental health and substance use prevention programs that had been evaluated in randomized or cluster randomized controlled trials (RCTs/cRCTs). Using the unique intervention program as the unit of analysis, this review described program characteristics, identified intervention components, and examined how mental health promotion and substance use prevention components were combined within individual programs.

Therefore, this review aimed to systematically identify school-based integrated mental health and substance use prevention programs evaluated in randomized or cluster randomized controlled trials (RCTs/cRCTs) and to: (1) describe the characteristics of school-based integrated mental health and substance use prevention programs for adolescents, including their prevention strategies, delivery modes, providers, and duration; (2) identify the mental health promotion and substance use prevention components incorporated in these programs; and (3) descriptively examine how components from the two domains were combined within individual programs. In this review, program characteristics referred to structural and implementation-related features, whereas intervention components referred to the specific mental health promotion and substance use prevention content delivered within each program. Delivery mode described how an intervention was delivered, while prevention strategy described whether it targeted a universal population, a selectively identified group, or both. By examining these characteristics and cross-domain component combinations, this review sought to clarify the content and organization of the included programs and identify areas that remain underrepresented in the existing evidence base.

## 2. Materials and Methods

### 2.1. Study Design

Consistent with the review objectives, this review used the unique intervention program as the unit of analysis to describe program characteristics and intervention components and to descriptively examine how mental health promotion and substance use prevention components were combined within programs. Program characteristics referred to structural and implementation-related features, whereas intervention components referred to the specific mental health promotion and substance use prevention contents delivered within each program.

This review systematically identified randomized and cluster randomized controlled trials evaluating school-based integrated mental health and substance use prevention programs for adolescents. The review procedures were informed by established principles for transparent literature searching, predefined eligibility criteria, independent study selection, and structured reporting [29,30]. The unique intervention program was used as the unit of analysis to map program characteristics, intervention components, and cross-domain component combinations. Because the objective of this review was to characterize program features rather than evaluate intervention effectiveness, effectiveness outcomes were not extracted or synthesized. All eligible programs meeting the predefined inclusion criteria were included in the mapping regardless of their reported outcomes.

### 2.2. Identification and Selection of Studies

Studies included in this review were identified through a systematic literature search conducted for school-based integrated mental health and substance use prevention programs for adolescents. The review protocol was prospectively registered in the International Prospective Register of Systematic Reviews (PROSPERO; CRD420261374182). Electronic database searches were conducted in April 2026 using PubMed, Embase, Cochrane Library, CINAHL, PsycINFO, KMbase, KoreaMed, ScienceON, DBpia, KISS, RISS, and ProQuest Dissertations and Theses (PQDT). PQDT was included as a supplementary search source to identify potentially relevant trial records and corresponding peer-reviewed journal publications that might otherwise have been missed; however, dissertation and thesis records themselves were not eligible, and only peer-reviewed journal articles that met the predefined eligibility criteria were included in the final review. Studies published between January 2016 and April 2026 were considered for inclusion. The 2016 start date was prespecified in the original review to focus on contemporary integrated prevention programs evaluated during the preceding decade.

The search strategy was developed using four key concepts: adolescents, school-based settings, substance use, and mental health promotion or prevention. Both controlled vocabulary terms and free-text keywords were used. Search terms included combinations of keywords such as “adolescent,” “youth,” “school-based,” “substance use,” “alcohol,” “tobacco,” “cannabis,” “mental health,” “depression,” “anxiety,” “resilience,” “help-seeking,” “prevention,” and “intervention.” Boolean operators (“AND” and “OR”) were applied appropriately. The detailed search strategy is provided in Supplementary Table S1.

**Figure 1:**
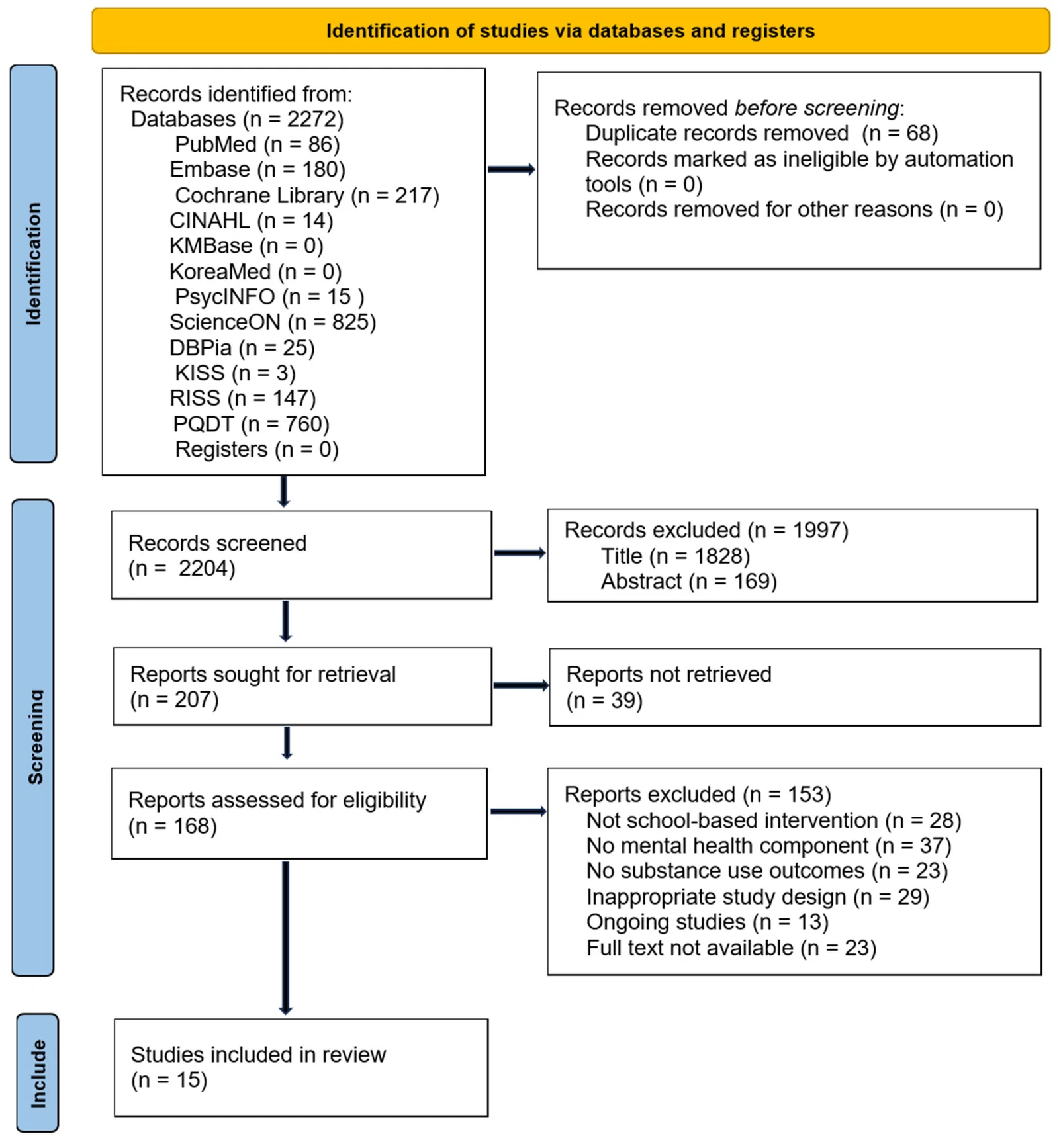
PRISMA flow of study selection process.

Following duplicate removal, titles and abstracts were screened independently by two reviewers. Potentially eligible studies underwent full-text review according to predefined eligibility criteria. Disagreements during study selection were resolved through discussion and consensus. The study selection process is summarized in Figure 1. A total of 15 studies representing 10 unique intervention programs met the predefined eligibility criteria and were included in the present review. Because multiple publications reported follow-up or secondary analyses of the same intervention, program characteristics were synthesized at the unique-program level. All eligible programs identified through the study selection process were included regardless of their reported intervention outcomes.

### 2.3. Eligibility Criteria

- Population:

The population of interest consisted of adolescents aged 13–18 years enrolled in middle schools, secondary schools, or high schools.

- Concept:

The review focused on school-based prevention programs addressing both substance use and mental health or psychosocial well-being. Mental health promotion components were operationally defined as intervention elements whose primary stated purpose, as described by the original study authors, was to improve mental health or psychosocial well-being, rather than to provide general life-skills training alone. For eligibility, programs were required to include at least one such broadly defined element in addition to a substance use prevention element. This requirement was used solely to establish that a program addressed both domains and was not used to preselect any specific mental health promotion component type. No prespecified mental health promotion component category was used to determine eligibility. Specific mental health promotion components were identified and coded during the data extraction, coding and analysis process.

- Context:

The context of interest included educational settings such as middle schools, secondary schools, and high schools. Studies were included if they:

1. Evaluated a school-based prevention program addressing both substance use and mental health or psychosocial well-being.
2. Included adolescent participants aged 13–18 years.
3. Reported substance use-related and/or mental health-related outcomes.
4. Employed a randomized controlled trial (RCT) or cluster randomized controlled trial (cRCT) design.
5. Were published in peer-reviewed journals in English.

Studies were excluded if they:

1. Focused exclusively on substance use prevention without an intervention element explicitly intended to promote mental health or psychosocial well-being.
2. Focused exclusively on mental health promotion without addressing substance use prevention.
3. Targeted university students or adults.
4. Evaluated treatment rather than prevention interventions.
5. Were conference abstracts, editorials, commentaries, reviews, dissertations, or protocols.
6. Were conducted outside school settings.

Because the objective of this review was to characterize school-based integrated prevention programs that had been evaluated using rigorous experimental designs, only randomized controlled trials (RCTs) and cluster randomized controlled trials (cRCTs) were included. This approach enabled a consistent description of programs that had undergone randomized evaluation. Consequently, implementation-oriented, pilot, quasi-experimental, and practice-based programs that had not undergone randomized evaluation were not represented in this review. Therefore, the findings should be interpreted as a descriptive map of integrated prevention programs evaluated in randomized trials rather than a comprehensive representation of all school-based integrated prevention programs.

### 2.4. Data Extraction and Coding

Data extraction and coding were conducted independently by two reviewers using a standardized data extraction and coding form developed for this review. Extracted information included:

- Author and year of publication;
- Country;
- Sample characteristics;
- Program name;
- Prevention strategy (universal, selective, or combined);
- Mental health promotion components;
- Substance use prevention components;
- Delivery mode;
- Intervention provider;
- Intervention duration;
- Additional program components outside the mental health promotion and substance use prevention domains;
- The original program description or stated purpose supporting each component code;
- The source used for each coding decision, including the primary study, protocol, intervention manual, or supplementary material, where available;
- The rationale for coding borderline or potentially overlapping elements.

Mental health promotion and substance use prevention components were independently coded by the two reviewers at the program level. Coding was based on the stated purpose and intervention content described by the original study authors. Evidence for coding was drawn from descriptions in the primary study reports and, where available, related protocols, intervention manuals, and Supplementary Materials. Operational definitions specified the purpose and content required for each code and the decision rules used to distinguish closely related categories (Supplementary Tables S2 and S3). A component was coded only when the program description provided explicit evidence consistent with the relevant operational definition; a broad label or presumed theoretical relevance alone was insufficient. When a single element explicitly served both mental health promotion and substance use prevention functions, codes in both domains were permitted. However, peer involvement, family contact, group participation, or provider type alone was treated as a delivery or implementation characteristic unless structured content meeting the operational definition of peer support or a family-focused approach was described. An intervention element was coded as a mental health promotion component only when its primary stated purpose was to improve mental health or psychosocial well-being, rather than to provide general life-skills training alone. Elements were coded as substance use prevention components when they were explicitly intended to influence substance-related knowledge, risk perceptions, beliefs, intentions, resistance or refusal skills, harm-reduction practices, help-seeking, or substance use behavior. General life-skills content was not coded within either focal domain unless the original program description explicitly linked that content to a mental health promotion or substance use prevention purpose. Similarly, provider type, peer involvement, family contact, and delivery format were treated as delivery or implementation characteristics unless the program included structured content explicitly intended to promote peer support, family support, mental health, or substance use prevention. Programs with multiple or overlapping aims were coded for all applicable component codes because the codes were not mutually exclusive. The same intervention element could receive codes in both domains when the source material explicitly supported both a mental health promotion function and a substance use prevention function. Conceptual relevance to both domains, without an explicitly described purpose or content, was not sufficient for dual coding. No predominant component was assigned. Additional program components outside the two focal domains were recorded separately based on the aims and content reported by the original study authors. For borderline or ambiguous elements, the reviewers compared the stated purpose, targeted outcome, intervention activities, and context of delivery against the operational definitions. The final decision and its rationale were documented in the program-level data extraction and coding form. Any discrepancies arising during data extraction or coding were resolved through discussion and consensus.

### 2.5. Data Analysis

Extracted and coded data were synthesized using descriptive statistics and descriptive narrative synthesis. Frequencies and percentages were calculated at the unique-program level to summarize prevention strategies, delivery modes, mental health promotion components, and substance use prevention components.

Because multiple publications reported secondary analyses or follow-up outcomes from the same intervention program, data were synthesized at the program level rather than the study level. This approach enabled the identification of unique prevention programs while minimizing duplication arising from multiple reports of the same intervention. The 10 unique programs were therefore used as the denominator for program-level frequency calculations.

Following individual component coding, mental health promotion components were used as anchors for a descriptive cross-domain map because the purpose of the mapping was to illustrate how mental health promotion elements were integrated with substance use prevention components within individual programs.

Closely related components were grouped solely to improve readability. Emotional regulation, coping skills, and cognitive behavioral strategies were displayed under self-regulation, whereas communication, social, and decision-making skills were displayed under social competence. These broader display headings were developed after coding, were not prespecified before study review, and were not derived from a particular theoretical framework. The original component codes were retained, and these display groupings were not used to classify programs or determine eligibility. No predominant component was assigned, and programs were not placed into mutually exclusive categories. Substance use prevention components identified within the same programs were then examined descriptively in relation to each mental health promotion component or grouping. Programs could contribute to multiple descriptive categories because all applicable components were retained. Program representation within each descriptive category was reported as the number of contributing programs among the 10 unique included programs (*n*/10).

Because the objective of this review was to characterize program features rather than evaluate intervention effectiveness, effectiveness outcomes and risk of bias were not synthesized. The analysis focused on describing program characteristics, intervention components, and cross-domain component patterns across the included programs.

## 3. Results

### 3.1. Characteristics of Included Programs

A total of 15 studies met the eligibility criteria and were included in this review (Figure 1). Because several publications reported secondary analyses or long-term follow-up outcomes derived from the same intervention program, intervention characteristics were synthesized at the program level. Consequently, the 15 included studies represented 10 unique intervention programs. The included programs were conducted predominantly in Australia, with additional programs identified in Switzerland and Brazil. Detailed characteristics of the included programs are presented in Table 1. Additional program-level information on intervention duration and dose, control conditions, and maximum follow-up period is provided in Supplementary Table S4.

Universal prevention was the predominant prevention strategy across programs, whereas selective prevention approaches were less common. In the included studies, selective prevention was implemented through personality-targeted interventions in which adolescents were selected based on validated personality risk profiles. Combined universal–selective approaches were identified in a small number of programs that integrated school-wide and targeted prevention components.

Regarding delivery methods, programs used web-based platforms, mobile applications, classroom-based sessions, or combinations of digital and face-to-face formats. Most programs were delivered by teachers, teacher-supervised facilitators, or trained school personnel. Psychologists were involved primarily in programs employing selective prevention approaches. Family engagement components were identified in one program, whereas peer-support approaches were identified in two programs.

The included programs were also examined for components outside the mental health promotion and substance use prevention domains. In Climate Schools Plus, the parent-directed content represented a family-focused mental health promotion approach combined with parent-mediated substance use prevention strategies, rather than a separate intervention domain. No distinct additional component outside the two focal domains was identified.

### 3.2. Mental Health Promotion Components

The mental health promotion components identified across the included programs are summarized in Table 2. Mental health literacy was the most frequently identified mental health promotion component, appearing in five of the ten intervention programs (50.0%). Emotional regulation was identified in four programs (40.0%). Help-seeking promotion, coping skills, cognitive behavioral strategies, and communication skills were each identified in three programs (30.0%). Social skills, decision-making skills, and peer support were identified in two programs (20.0%). Family-focused approaches were the least frequently identified component, appearing in one program (10.0%).

Overall, the findings indicate that the integrated prevention programs included in this review incorporated multiple mental health promotion components alongside substance use prevention content, particularly mental health literacy, emotional regulation, coping, and help-seeking components.

### 3.3. Substance Use Prevention Components

The substance use prevention components identified across the included programs are presented in Table 3. Drug literacy and resistance skills were the most frequently identified substance use prevention components, each appearing in six of the ten intervention programs (60.0%). These components primarily focused on increasing knowledge about substance use and strengthening adolescents’ ability to resist peer pressure and substance-related risk situations. Alcohol prevention was identified in five programs (50.0%), followed by cannabis prevention in four programs (40.0%). Harm reduction strategies were incorporated in three programs (30.0%), tobacco prevention, substance-use help-seeking, and substance-use risk reduction were each identified in two programs (20.0%). These components were typically incorporated alongside broader prevention strategies rather than being addressed as standalone intervention targets.

### 3.4. Descriptive Cross-Domain Analysis of Mental Health Promotion and Substance Use Prevention Components

To examine how components from the two domains were combined, mental health promotion components were used as anchors for program-level cross-domain descriptive analysis (Table 4). For presentation purposes, closely related components were organized into six non-exclusive descriptive categories: mental health literacy; self-regulation, comprising emotional regulation, coping skills, and cognitive behavioral strategies; help-seeking promotion; social competence, comprising communication, social, and decision-making skills; peer support; and a family-focused approach.

The mental health literacy descriptive category was represented in 5 programs, self-regulation in 4, help-seeking promotion and social competence in 3 programs each, peer support in 2, and the family-focused approach in 1. Programs could contribute to more than one descriptive category because all applicable components were retained.

Among the 5 programs incorporating mental health literacy, drug literacy co-occurred in 4 programs and resistance skills in 3. Harm reduction and substance-use help-seeking each co-occurred with mental health literacy in 2 programs, whereas alcohol and cannabis prevention each co-occurred in 1 program.

Self-regulation components were identified in 4 programs. Emotional regulation was present in all 4, while coping skills and cognitive behavioral strategies were each present in 3. Alcohol and cannabis prevention each co-occurred with self-regulation components in 3 programs. In both programs incorporating substance-use risk reduction—Climate and Preventure and Preventure—this component was combined with emotional regulation, coping skills, and cognitive behavioral strategies.

Help-seeking promotion was identified in 3 programs. It co-occurred with substance-use help-seeking and drug literacy in 2 programs each, while resistance skills and harm reduction each occurred in 1.

Social-competence components were also identified in 3 programs. Communication skills were present in all 3, while social and decision-making skills were each present in 2. SmartCoach and #Tamojunto 2.0 both combined communication, social, and decision-making skills with resistance skills, alcohol prevention, and tobacco prevention. Mind Your Mate combined communication skills with drug literacy and substance-use help-seeking.

Peer support was identified in 2 programs, The Illicit Project and Mind Your Mate. Both programs also incorporated mental health literacy, help-seeking promotion, and drug literacy. The Illicit Project additionally included resistance skills and harm reduction, whereas Mind Your Mate included communication skills and substance-use help-seeking.

A family-focused approach was identified only in Climate Schools Plus and was combined with drug literacy, resistance skills, alcohol prevention, cannabis prevention, and harm reduction.

Multiple mental health promotion components or display groupings were identified within individual programs. Climate Schools Combined incorporated mental health literacy together with self-regulation components; SmartCoach incorporated both self-regulation and social-competence components; and The Illicit Project and Mind Your Mate each incorporated mental health literacy, help-seeking promotion, and peer support. Accordingly, all applicable component configurations were retained in the mapping rather than assigning each program to a single category. Overall, no single cross-domain component configuration was consistently represented across the majority of programs, and several descriptive categories and component combinations were identified in only one or two programs.

## 4. Discussion

This review systematically identified and described the characteristics of school-based integrated mental health and substance use prevention programs evaluated in randomized and cluster randomized controlled trials. Fifteen studies representing 10 unique intervention programs were included. Mental health literacy, drug literacy, and resistance skills were the most frequently identified components, while considerable diversity was observed in how mental health promotion and substance use prevention components were combined across programs. These findings provide an overview of the characteristics of currently evaluated integrated prevention programs rather than a comprehensive representation of all school-based integrated prevention initiatives.

This review has several strengths. It systematically described the characteristics, prevention strategies, delivery approaches, and cross-domain component patterns of school-based programs integrating mental health promotion and substance use prevention for adolescents. By focusing on programs evaluated in randomized controlled trials and cluster randomized controlled trials, this review provides a synthesis of the characteristics of integrated school-based prevention programs.

A further strength of this review is the use of program-level synthesis. Because several publications reported secondary analyses or long-term follow-up outcomes from the same intervention program, the review synthesized findings across 10 unique intervention programs rather than treating all studies as independent interventions. This approach reduced duplication and enabled a clearer understanding of the characteristics and component configurations of the included programs.

Another strength is the use of a non-exclusive cross-domain component map. Rather than assigning multidimensional programs to mutually exclusive types or identifying a predominant component, the map retained all applicable component codes and displayed how mental health promotion and substance use prevention components co-occurred across programs. This approach also distinguished intervention content from delivery modes and implementation characteristics.

Several limitations should also be acknowledged. First, although a comprehensive literature search was conducted across multiple electronic databases, the review was limited to studies identified within the predefined search strategy and eligibility criteria. Additional relevant studies published outside the search period or not meeting these criteria may therefore not have been captured. The findings should therefore be interpreted as a descriptive synthesis of this defined evidence base rather than an exhaustive account of all integrated programs implemented in schools.

Second, only randomized controlled trials and cluster randomized controlled trials were included. While this decision ensured that the included programs had been examined using experimental designs, potentially relevant programs evaluated using alternative study designs were excluded. Consequently, the review may underrepresent emerging, locally developed, or practice-based programs that have not undergone randomized evaluation. The characteristics of the included programs may therefore differ from those of integrated programs implemented in routine educational practice.

Third, the programs varied in intervention content, prevention strategy, delivery mode, provider, and implementation characteristics. In addition, component coding depended on the aims and intervention descriptions reported by the original study authors. Incomplete or inconsistent reporting may have resulted in some components being overlooked or coded less precisely. Although disagreements were resolved through reviewer discussion and consensus, some interpretive judgement remained unavoidable.

Fourth, most included programs were developed or evaluated in Australia, with only a small number conducted in other countries. The identified component patterns and delivery characteristics may therefore reflect the educational structures, prevention priorities, service systems, and technological resources of a geographically concentrated evidence base. Transferability to other cultural and educational contexts should not be assumed, particularly in lower-resource settings where reliable digital infrastructure, trained facilitators, specialist mental health services, or established referral systems may be less available. Cultural differences in attitudes toward mental health, substance use, help-seeking, family involvement, and school responsibilities may also influence program acceptability and implementation.

Finally, because this review was intended to characterize program features rather than evaluate comparative effectiveness, a formal risk-of-bias assessment was not conducted. Although all included programs were evaluated using randomized designs, differences in methodological quality and effectiveness cannot be inferred from the present review.

The frequency of a component, its position in the cross-domain map, or its co-occurrence with other components does not indicate that it is effective or superior. Because the map was derived from only 10 unique programs, it should be interpreted as a descriptive representation of the included evidence rather than as a validated or comprehensive framework. The overlapping descriptive categories should not be summed or used to classify programs into fixed categories. Future systematic reviews should formally assess risk of bias and the certainty of evidence when sufficient comparable studies become available. Further rigorously designed trials, independent replication, and longer-term follow-up are also needed to determine whether particular components or combinations are associated with effectiveness, feasibility, equity, and sustainability across different school contexts.

### 4.1. Principal Findings

A central finding was that the included programs combined traditional substance use prevention approaches, including substance-related education and harm reduction [15,16], with mental health promotion components such as mental health literacy, emotional regulation, coping skills, cognitive behavioral strategies, communication skills, peer support, and help-seeking promotion [19,20,27]. Rather than indicating a transition shared by school-based prevention programs generally, this pattern describes how integration was operationalized within the programs included in this review. The co-occurrence of components from both domains is conceptually consistent with evidence that adolescent substance use and mental health problems can co-occur and share developmental and psychosocial risk and protective factors [8,9,10].

The present review extends the previous literature by focusing on how integrated school-based prevention programs have been designed rather than on whether they are effective. The findings indicate that integrated programs most commonly combined mental health literacy, emotional regulation, coping skills, and help-seeking promotion with substance use prevention components such as drug literacy, resistance skills, and alcohol or cannabis prevention. However, considerable variation was observed in the combinations of components across programs, suggesting that no single model has yet emerged as the dominant approach. The cross-domain descriptive analysis developed in this review provides a descriptive framework for understanding how mental health promotion and substance use prevention have been integrated within existing randomized school-based programs. Because this review did not evaluate comparative effectiveness, the identified component configurations should not be interpreted as indicating superiority or effectiveness of any particular program or combination of components.

A key contribution of the present review is that it systematically examined how school-based integrated prevention programs have been designed, delivered, and implemented across randomized evaluations. The review also developed a descriptive cross-domain component map illustrating how mental health promotion components co-occurred with substance use prevention components within individual programs. Rather than classifying programs into mutually exclusive categories, the map retains overlapping component configurations and distinguishes intervention content from delivery and implementation characteristics. This approach better reflects the multidimensional nature of integrated prevention programs. However, because the map was derived from only 10 unique programs, it should be regarded as a descriptive synthesis of the available evidence rather than a validated classification framework.

The cross-domain analysis also identified considerable diversity in how mental health promotion and substance use prevention components were combined across the 10 unique programs. No single cross-domain component configuration predominated, and some component groupings, particularly peer support and the family-focused approach, were represented in only one or two programs. This diversity illustrates the range of approaches being explored but also indicates limited replication of specific component configurations. Consequently, the current evidence does not permit conclusions regarding which configurations provide the most reliable basis for broader implementation. Independent replication, adequately powered trials, longer-term follow-up, and evaluations across diverse cultural and educational contexts are needed before specific program approaches can be recommended for wider implementation.

Universal prevention was frequently used among the included programs [26,38], which is consistent with the capacity of school-based approaches to reach students without requiring individual risk identification. Selective programs in which participants were identified based on validated personality risk profiles and programs combining universal and selective elements were also identified. These approaches illustrate alternative ways of matching the intensity or content of prevention to students’ risk profiles. However, their presence within this small group of programs does not suggest a broader shift in prevention practice or indicate that one prevention strategy is preferable to another.

Digital, web-based, mobile, and blended delivery formats were used in several of the included programs. Personality-targeted selective interventions represented an alternative participant selection strategy, in which adolescents were identified based on validated personality risk profiles, within the included evidence [35]. Although technology-supported interventions may offer potential advantages in scalability, accessibility, implementation fidelity, and cost-effectiveness [35], these advantages cannot be assumed across all school systems. Their implementation depends on access to appropriate devices and internet infrastructure, staff training, technical support, data protection procedures, and available referral pathways. These requirements may limit transferability to schools in lower-resource settings or settings with limited access to mental health professionals.

Mental health literacy and help-seeking promotion were incorporated into programs such as MAKINGtheLINK and Mind Your Mate, which encouraged adolescents to recognize mental health concerns, support peers, and access appropriate professional assistance [27,33]. These components may be relevant to persistent gaps between adolescents’ mental health needs and service utilization. However, their practical value is likely to depend on whether schools and communities provide accessible, confidential, and responsive support and referral services. Accordingly, help-seeking promotion should be considered in conjunction with the service capacity of the settings in which programs are implemented.

### 4.2. Mental Health Promotion Components in Integrated Programs

The identified programs consistently integrated mental health promotion with substance use prevention by incorporating components that addressed psychological well-being, emotional regulation, adaptive coping, and access to support. This pattern suggests that integrated school-based prevention has increasingly focused on shared psychosocial mechanisms underlying both mental health problems and substance use, rather than addressing these issues as separate targets. Given that many adolescents experiencing psychological distress do not receive timely or appropriate support [1], incorporating these components into school-based prevention programs may help strengthen early recognition and support pathways. Nevertheless, the present review does not indicate that any particular component is more important or more effective than another.

Mental health literacy may facilitate early identification of mental health concerns, improve awareness of available resources, reduce stigma, and encourage timely access to professional services. The inclusion of mental health literacy within integrated prevention programs reflects increasing recognition that improving adolescents’ understanding of mental health may complement efforts to prevent substance use by facilitating early recognition of problems and encouraging appropriate help-seeking [27,33]. Nevertheless, the potential benefit of mental health literacy and help-seeking content depends partly on the availability of confidential, accessible, and responsive support services. Increasing recognition of mental health concerns without ensuring appropriate referral and follow-up pathways may place additional demands on teachers and schools and may not result in timely access to care. Previous research has shown that difficulties in emotional regulation and maladaptive coping strategies are associated with increased vulnerability to substance use, risk-taking behaviors, and psychological distress [9,10,13]. This shared relevance provides a conceptual rationale for including emotional regulation and adaptive coping in integrated programs. Although these components provide a plausible rationale for integrated prevention, their individual contributions to program outcomes remain unclear. Several programs incorporated emotional regulation, coping skills, and cognitive behavioral strategies, reflecting an emphasis on shared psychosocial mechanisms underlying both mental health and substance use [34,36,37]. Help-seeking promotion represented another important approach to integrating mental health promotion with substance use prevention [27,33]. Whether these components contribute to improved mental health or substance use outcomes remains an important question for future research. Further research should examine whether specific components or combinations contribute to mental health and substance use outcomes and whether their effects vary according to student characteristics and implementation context.

Taken together, the findings show that the included programs operationalized integration through multiple, overlapping mental health promotion components. The cross-domain map provides a useful framework for understanding how mental health promotion and substance use prevention have been integrated within existing school-based programs and may inform the design of future integrated interventions. Given the small number and geographic concentration of the included programs, broader conclusions about current school-based prevention practice should be made cautiously.

### 4.3. Delivery Characteristics and Implementation Considerations

The diversity of prevention strategies identified across the included programs suggests that integrated school-based prevention can be adapted to different levels of student need and educational contexts. Universal delivery can reach students without requiring individual risk identification and may offer potential advantages in accessibility and stigma reduction [23]. However, its feasibility depends on whether schools can allocate sufficient curriculum time, train staff, and maintain implementation fidelity across large groups of students. The selective and combined approaches identified in this review illustrate how additional support may be directed toward adolescents with particular vulnerability profiles. Such approaches may require validated screening procedures, safeguards against labelling and stigma, and access to professionals capable of providing more intensive intervention.

Several included programs used web-based platforms, mobile applications, or blended formats combining digital and face-to-face delivery [20,37,38]. Digital delivery may improve accessibility and facilitate standardized implementation under some conditions [39]. Nevertheless, the use of technology does not necessarily reduce all implementation barriers. Digital programs require reliable internet access, appropriate devices, technical support, data protection procedures, and sufficient digital literacy among students and staff. These requirements may be difficult to meet in lower-resource schools and could exacerbate disparities if digital access is uneven. Low-bandwidth, offline, or teacher-led alternatives may therefore be necessary when adapting these programs to different educational contexts.

Teachers, teacher-supervised facilitators, and trained school personnel were involved in delivering many of the programs. Using existing school personnel may support integration into educational structures, but it also creates practical demands related to training, preparation time, workload, confidence in discussing sensitive topics, and responding to student disclosures. Programs requiring psychologists or specialized facilitators may face additional workforce and cost constraints. These implementation requirements are particularly relevant in school systems with limited access to mental health professionals or weak referral pathways.

Some programs extended intervention activities beyond classroom-based learning by incorporating parent-focused or peer-supported components. These approaches may strengthen interpersonal support and address social contexts relevant to adolescent mental health and substance use. Their implementation, however, requires sustained parent engagement, appropriate preparation and supervision of peer supporters, clearly defined role boundaries, and procedures for managing confidentiality and risk [33,38]. Future research should evaluate whether family- and peer-oriented components enhance program implementation, sustainability, and outcomes across different school settings.

Taken together, the review identified diverse delivery strategies among the included integrated school-based prevention programs rather than a single standardized model. Selection of delivery approaches should therefore consider local educational infrastructure, workforce capacity, cultural context, referral systems, and student needs. Future implementation research across diverse school settings is needed to evaluate the feasibility, acceptability, sustainability, and scalability of these approaches.

### 4.4. Implications for Research and Practice

The findings of this review provide several implications for researchers, educators, healthcare professionals, and policymakers involved in adolescent health promotion. The identified component patterns may inform the design, implementation, and evaluation of future integrated school-based prevention programs.

First, the findings support the continued development and rigorous evaluation of prevention programs that address mental health promotion and substance use prevention together. Growing evidence suggests that these outcomes share several common risk and protective factors, including emotional dysregulation, maladaptive coping, impulsivity, peer influences, and barriers to help-seeking [8,9,10]. Interventions targeting these shared mechanisms may therefore have the potential to influence both domains simultaneously [14]. Future comparative studies should examine whether targeting shared psychosocial mechanisms is associated with additional benefits compared with single-domain interventions for mental health, substance use, or both.

Second, the component patterns identified in this review may inform the design and evaluation of future integrated programs. Although strengthening psychosocial competencies may contribute to both mental health promotion and substance use prevention [18,21,22], the relative contribution of individual components and their combinations remains unclear. Future studies should use component-level analyses, factorial designs, or other comparative approaches to identify which intervention components, alone or in combination, contribute most to program outcomes.

Third, the use of web-based platforms, mobile applications, and blended learning approaches within the included programs highlights the potential for technology-supported delivery [31,33,37]. Nevertheless, scalability should not be assumed from delivery format alone. Digital implementation requires appropriate infrastructure, device and internet access, staff preparation, technical support, data protection, and procedures for responding to students who disclose mental health or substance use concerns. Schools with limited access to mental health professionals may face particular challenges because digital identification or help-seeking activities can increase demand for services without increasing referral capacity. Implementation studies should therefore assess resource requirements, equity of access, fidelity, cost, acceptability, and linkage to appropriate support services.

Fourth, the cross-domain descriptive framework provides a structured framework for describing how mental health promotion and substance use prevention components are integrated within school-based programs. Although derived from a limited number of programs, it may facilitate transparent reporting, comparison across interventions, and the identification of component configurations for future investigation.

Future research should prioritize consistent operational definitions and detailed reporting of intervention components rather than prematurely establishing fixed classification systems. Studies should also report provider qualifications, training requirements, implementation fidelity, adaptations, costs, referral arrangements, and contextual conditions. Greater inclusion of programs from diverse cultural, educational, and socioeconomic settings is needed to determine whether the component combinations identified here are feasible and relevant beyond the predominantly Australian evidence base. In lower-resource settings, adaptation may require low-technology delivery options, greater reliance on existing school personnel, and stronger coordination with available community services. Such adaptations should be evaluated to determine whether they preserve the intended functions of the intervention components.

## 5. Conclusions

This review described the characteristics and intervention components of 10 school-based integrated mental health and substance use prevention programs evaluated in 15 randomized or cluster randomized controlled trials. The cross-domain descriptive analysis identified the diverse ways in which mental health promotion and substance use prevention components have been integrated within existing school-based programs and provides a descriptive framework for comparing future interventions. Because the available evidence was limited and geographically concentrated, the findings should be interpreted within the context of the included programs. Future comparative, component-level, and implementation research across diverse cultural, educational, and resource settings is needed to evaluate the effectiveness, feasibility, equity, and sustainability of these approaches to integrated school-based prevention.

## Figures and Tables

**Table 1:** Characteristics of Included Intervention Programs (*n* = 10).

Program	Country	Prevention Strategy	Delivery Mode	Provider
Climate Schools: Ecstasy and Emerging Drugs	Australia	Universal	Web-based	Teacher
Climate and Preventure (CAP)	Australia	Combined universal–selective	Web-based and face-to-face	Teacher and psychologist
MAKINGtheLINK	Australia	Universal	Classroom-based	Teacher
Climate Schools Combined (CSC)	Australia	Universal	Web-based	Teacher
Preventure	Australia	Selective	Face-to-face	Psychologist
SmartCoach	Switzerland	Universal	Mobile- and web-based	Automated digital platform
The Illicit Project	Australia	Universal	Classroom-based	Teacher
#Tamojunto 2.0	Brazil	Universal	Classroom-based	Teacher
Climate Schools Plus	Australia	Universal	Online student and parent program	Teacher and parent
Mind Your Mate	Australia	Universal	Web-based and mobile application	Teacher-supported digital program

**Table 2:** Mental Health Promotion Components Across Included Programs (*n* = 10).

Mental Health Promotion Component	*n* (%)
Mental Health Literacy	5 (50.0)
Emotional Regulation	4 (40.0)
Help-Seeking Promotion	3 (30.0)
Coping Skills	3 (30.0)
Cognitive Behavioral Strategies	3 (30.0)
Communication Skills	3 (30.0)
Social Skills	2 (20.0)
Decision-Making Skills	2 (20.0)
Peer Support	2 (20.0)
Family-Focused Approaches	1 (10.0)

**Table 3:** Substance Use Prevention Components Across Included Programs (*n* = 10).

Substance Use Prevention Component	*n* (%)
Drug Literacy	6 (60.0)
Resistance Skills	6 (60.0)
Alcohol Prevention	5 (50.0)
Cannabis Prevention	4 (40.0)
Harm Reduction	3 (30.0)
Tobacco Prevention	2 (20.0)
Substance-Use Help-Seeking	2 (20.0)
Substance-Use Risk Reduction	2 (20.0)

**Table 4:** Program-Level Descriptive Summary of Mental Health Promotion and Substance Use Prevention Components Across Included Programs (*N* = 10).

Program Name	Intended or Implemented Age Range (School Year/Grade)	Mental Health Promotion Components Assigned	Substance Use Prevention Components Assigned
Climate Schools: Ecstasy and Emerging Drugs [31]	14–15 years (Year 10)	Mental health literacy	Drug literacy, Resistance skills, Harm reduction, Alcohol prevention, Cannabis prevention
MAKINGtheLINK [27]	14–15 years (Year 9)	Mental health literacy, Help-seeking promotion	Drug literacy, Resistance skills, Harm reduction, Substance-use help-seeking
Climate Schools Combined [19]	13.5–16.0 years (middle school)	Mental health literacy, Emotional regulation, Coping skills, Cognitive behavioral strategies	Drug literacy, Resistance skills, Alcohol prevention, Cannabis prevention
The Illicit Project [32]	15–19 years (Years 10–12)	Mental health literacy, Help-seeking promotion, Peer support	Drug literacy, Resistance skills, Harm reduction, Substance-use help-seeking
Mind Your Mate [33]	Approximately 14–15 years (Year 9)	Mental health literacy, Help-seeking promotion, Communication skills, Peer support	Drug literacy, Resistance skills, Harm reduction, Substance-use help-seeking
Climate and Preventure [19,34,35]	Approximately 13–14 years (Year 8)	Emotional regulation, Coping skills, Cognitive behavioral strategies	Drug literacy, Resistance skills, Alcohol prevention, Cannabis prevention
Preventure [35,36]	13–14 years (Year 8)	Emotional regulation, Coping skills, Cognitive behavioral strategies	Alcohol prevention, Cannabis prevention, Substance-use risk reduction, Tobacco prevention
SmartCoach [37]	14–17 years (secondary and upper-secondary school students)	Emotional regulation, Decision-making skills	Alcohol prevention, Tobacco prevention
#Tamojunto 2.0 [28]	Approximately 12–14 years (Grade 8)	Communication skills, Social skills, Decision-making skills	Drug literacy, Resistance skills, Alcohol prevention, Tobacco prevention, Cannabis prevention
Climate Schools Plus [38]	12–14 years at initiation (Year 8; continued in Year 9)	Family-focused approach	Drug literacy, Resistance skills, Harm reduction, Alcohol prevention, Cannabis prevention

Note. Age ranges refer to the populations for whom the programs were intended or initially implemented; approximate ranges correspond to the reported school grades. Numbers in brackets indicate the included publication(s) for each intervention program. Components were coded non-exclusively at the program level based on primary reports, protocols, and manuals. Operational definitions and coding rules are provided in Supplementary Tables S2 and S3.

## Data Availability

The data presented in this study are available within the article and Supplementary Materials.

## Supplementary Materials

The following supporting information can be downloaded at https://www.mdpi.com/article/10.3390/children13091139/s1. Supplementary Table S1: Search Strategy (PubMed); Supplementary Table S2: Operational Definitions, Coding Criteria, and Decision Rules for Mental Health Promotion Components (Corresponds to Table 2); Supplementary Table S3: Operational Definitions, Coding Criteria, and Decision Rules for Substance Use Prevention Components (Corresponds to Table 3); Supplementary Table S4: Intervention Duration, Control Conditions, and Follow-Up of the Included Intervention Programs [40,41].
