# Supplementary material for: Review of the Characteristics of School-Based Integrated Mental Health and Substance Use Prevention Programs for Adolescents Evaluated Through Clinical Trials Between 2016 and 2026
Source: Children (Basel). 2026 Aug 25;13(9):1139. doi: 10.3390/children13091139 (PMC13605885; doi:10.3390/children13091139)
Supplement: Supplementary file 1 [file children-13-01139-s001.zip › children-4422705-supplementary.pdf]

Supplementary Table S1. Search Strategy (PubMed)

No.	Search term
1	Adolescent [Mesh]
2	Adolescent* OR Teen* OR Youth* OR "High school student*" OR "Middle school student*"
3	1 OR 2
4	Schools [Mesh]
5	“School-based” OR "School program*" OR "School intervention*" OR "Educational setting" OR Classroom*
6	4 OR 5
7	Substance-Related Disorders [Mesh]
8	"Substance use" OR "Drug use" OR "Substance abuse" OR Alcohol* OR Smoking OR Tobacco OR Vaping OR Cannabis OR Marijuana
9	7 OR 8
10	Mental Health [Mesh]
11	"Mental health" OR Depression OR Anxiety OR Stress OR Resilience OR "Psychological well-being" OR "Emotional well-being" OR "School adjustment" OR "Self-esteem"
12	10 OR 11
13	Prevention and Control[Mesh] OR Health Promotion [Mesh] OR Primary Prevention [Mesh]
14	Prevention OR Intervention* OR Program* OR "Preventive intervention*" OR "Health education"
15	13 OR 14
16	3 AND 6 AND 9 AND 12 AND 15

Supplementary Table S2. Operational Definitions, Coding Criteria, and Decision Rules for Mental Health Promotion Components (Corresponds to Table 2)

Component	Operational Definition	Required Evidence for Coding	Distinguishing Criteria / Decision Rules
Mental Health Literacy	Educational content aimed at increasing knowledge about mental health conditions, reducing stigma, or understanding psychosocial well-being.	Explicit description of educational modules or activities focused on mental health facts, symptom recognition, or anti-stigma concepts.	Must focus on general mental health and emotional well-being. Knowledge specifically about drugs, alcohol, or addiction is coded as Drug Literacy (Supplementary Table S4).
Emotional Regulation	Strategies and skills designed to recognize, understand, manage, and express emotions in adaptive ways.	Program content teaching techniques for identifying feelings, managing stress, or controlling emotional reactivity (e.g., mindfulness, relaxation).	Focuses specifically on internal emotional states and self-control, whereas Coping Skills deals with broader external stressors and problem-solving.
Help-Seeking Promotion	Structured instruction or guidance encouraging individuals to seek professional or informal support for personal or mental health issues.	Content providing resources, pathways, or skill-building to reach out to counselors, helplines, or trusted adults for mental health concerns.	Must target general mental health or emotional distress. Help-seeking specifically related to substance misuse is coded as Substance-Use Help-Seeking (Supplementary Table S4).
Coping Skills	Behavioral or cognitive strategies aimed at managing specific stressful situations, adversity, or daily life challenges.	Explicit teaching of stress-management tools, problem-solving frameworks, or adaptive coping mechanisms.	Focuses on managing external stressors/challenges, distinguished from Emotional Regulation which targets internal emotional states.

Cognitive Behavioral Strategies	Interventions using core cognitive-behavioral therapy (CBT) principles, such as identifying and restructuring negative thought patterns (cognitive reframing).	Description of activities targeting cognitive distortions, self-talk, or core CBT exercises (e.g., thought logs, cognitive restructuring).	Requires explicit CBT-based cognitive restructuring techniques, rather than general decision-making or knowledge transmission.
Communication Skills	Content explicitly aimed at enhancing interpersonal communication, active listening, or expressing thoughts and feelings effectively.	Structured modules or exercises dedicated to verbal/non-verbal communication techniques or assertive expression in general social contexts.	Must target general interpersonal communication. Refusal or resistance techniques specific to peer pressure around drug use are coded as Resistance Skills (Supplementary Table S4).
Social Skills	Broad interpersonal competencies designed to build and maintain healthy social relationships and group interactions.	Structured exercises or curriculum promoting perspective-taking, empathy, or prosocial relationship building.	Focuses on broader social relational dynamics, whereas Communication Skills focuses specifically on message exchange/expression techniques.
Decision-Making Skills	Structured frameworks teaching step-by-step problem-solving or choices regarding personal life choices and goals.	Teaching generic decision-making models (e.g., identifying options, evaluating consequences) for general lifestyle choices.	Focuses on general life choices. If decisions are explicitly linked to resisting substance offers, it is coded under Resistance Skills or Substance-Use Risk Reduction (Supplementary Table S4).
Peer Support	Structured program components designed to facilitate mutual support, peer mentoring, or guided	Active, structured peer-led activities, peer mentoring programs, or mutual help delivery formats.	Requires structured supportive content/roles; simple group administration or peer presence alone is

	peer-to-peer discussion groups.		treated as a delivery format, not Peer Support.
Family-Focused Approaches	Interventions directly involving family members or providing structured content to enhance parent-child communication and family functioning.	Program components requiring parent/caregiver involvement, family homework assignments, or parent training sessions.	Requires direct family participation or structured content targeting family dynamics; simple informational flyers sent home are insufficient.

Supplementary Table S3. Operational Definitions, Coding Criteria, and Decision Rules for Substance Use Prevention Components (Corresponds to Table 3)

Component	Operational Definition	Required Evidence for Coding	Distinguishing Criteria / Decision Rules
Drug Literacy	Educational content providing factual information about illicit drugs, psychoactive substances, pharmacology, or addiction mechanisms.	Explicit mention of teaching drug taxonomy, pharmacological effects, or addiction concepts.	Focuses on knowledge about drugs and substances; general mental health information is coded as Mental Health Literacy (Supplementary Table S3).
Resistance Skills	Specific skills training focused on recognizing peer pressure and practicing refusal strategies (e.g., "saying no" techniques).	Practical exercises, role-playing, or refusal assertive training explicitly directed at resisting drug or alcohol offers.	Must be specific to refusing substance use; general interpersonal skills are coded as Communication Skills (Supplementary Table S3).
Alcohol Prevention	Targeted educational or behavioral content specifically addressing the risks, consequences, or prevention of alcohol use.	Explicit focus on alcohol-specific knowledge, binge drinking risks, or alcohol misuse prevention.	Coded when alcohol is explicitly targeted, as distinct from generic drug education (Drug Literacy) or cannabis/tobacco specific content.

Cannabis Prevention	Targeted content specifically addressing the risks, legal aspects, health effects, or prevention of cannabis/marijuana use.	Explicit curriculum content or activities specifically focused on cannabis/marijuana misuse.	Explicitly targets cannabis, distinguishing it from general drug literacy or other specific substances.
Harm Reduction	Strategies aimed at minimizing the negative health, social, or physical consequences of potential or ongoing substance use.	Content teaching safer behaviors, harm minimization practices, or risk awareness without strictly requiring abstinence.	Focuses on minimizing harm/consequences of use, distinguished from Substance-Use Risk Reduction which targets lowering the likelihood of initiating use.
Tobacco Prevention	Targeted content specifically addressing the risks, health consequences, or prevention of tobacco and nicotine product use (including e-cigarettes/vaping).	Explicit modules or activities targeting smoking, vaping, or tobacco product prevention.	Specifically targets tobacco/nicotine, distinct from general drug education or other specific substances.
Substance-Use Help-Seeking	Specific guidance and pathways provided to assist individuals in obtaining help or treatment for substance misuse or addiction.	Content providing hotlines, referral paths, or skill-building specifically for seeking assistance for substance-related problems.	Must explicitly target substance misuse/addiction; general mental distress help-seeking is coded as Help-Seeking Promotion (Supplementary Table S3).
Substance-Use Risk Reduction	Behavioral or educational strategies targeted at identifying and avoiding specific high-risk triggers or environments associated with substance initiation or escalation.	Content identifying personal/environmental risk factors for substance use and teaching specific avoidance/mitigation steps.	Focuses on reducing exposure to risk factors/triggers prior to or during use, distinguished from Harm Reduction (minimizing consequences of use).

Supplementary Table S4. Intervention Duration, Control Conditions, and Follow-up of the Included Intervention Programs

Intervention Program	Key Publications	Intervention Duration and Dose	Control Condition	Maximum Follow-up
Climate Schools: Ecstasy and Emerging Drugs	Champion et al. (2016, 2018)	4 lessons × 40 min delivered over 4 weeks	Health education as usual	24 months
Climate and Preventure (CAP)	Teesson et al. (2017); Newton et al. (2018, 2022b)	12 universal lessons plus two 90-min selective sessions	Health education as usual	7 years
MAKINGtheLINK	Lubman et al. (2020)	Five classroom activities plus one booster session	Wait-list control	12 months
Climate Schools Combined (CSC)	Teesson et al. (2020); Newton et al. (2022a)	18 lessons (12 substance use; 6 mental health)	Active control / Health education as usual	30 months
Preventure	Debenham et al. (2021)	Two 90-min sessions	Health education as usual	36 months
SmartCoach	Haug et al. (2021)	22-week mobile/web-based intervention	Assessment-only control	18 months
The Illicit Project	Debenham et al. (2022)	Three classroom sessions	Health education as usual	6 months

#Tamojunto 2.0	Garcia-Cerde et al. (2023)	12 weekly lessons (50 min each)	No intervention	9 months
Climate Schools Plus	Slade et al. (2023)	12 student lessons plus parent modules	Health education as usual	24 months
Mind Your Mate	Birrell et al. (2023)	One classroom lesson plus mobile application	Health education as usual	12 months
